# Expression of Toll-Like Receptors in the Developing Brain

**DOI:** 10.1371/journal.pone.0037767

**Published:** 2012-05-30

**Authors:** David Kaul, Piet Habbel, Katja Derkow, Christina Krüger, Eleonora Franzoni, F. Gregory Wulczyn, Stefan Bereswill, Robert Nitsch, Eckart Schott, Rüdiger Veh, Thomas Naumann, Seija Lehnardt

**Affiliations:** 1 Department of Neurology, Charité-Universitaetsmedizin Berlin, Berlin, Germany; 2 Center for Anatomy, Institute of Cell Biology and Neurobiology, Charité-Universitaetsmedizin Berlin, Berlin, Germany; 3 Department of Microbiology and Hygiene, Charité-Universitaetsmedizin Berlin, Berlin, Germany; 4 Institute of Microscopic Anatomy and Neurobiology, Johannes Gutenberg-Universitaet Mainz, Mainz, Germany; 5 Department of Hepatology and Gastroenterology, Charité-Universitaetsmedizin Berlin, Berlin, Germany; 6 Cluster of Excellence NeuroCure, Charité-Universitaetsmedizin Berlin, Berlin, Germany; Centre d'Immunologie de Marseille-Luminy, CNRS-Inserm, France

## Abstract

Toll-like receptors (TLR) are key players of the innate and adaptive immune response in vertebrates. The original protein Toll in *Drosophila melanogaster* regulates both host defense and morphogenesis during development. Making use of real-time PCR, *in situ* hybridization, and immunohistochemistry we systematically examined the expression of TLR1–9 and the intracellular adaptor molecules MyD88 and TRIF during development of the mouse brain. Expression of TLR7 and TLR9 in the brain was strongly regulated during different embryonic, postnatal, and adult stages. In contrast, expression of TLR1–6, TLR8, MyD88, and TRIF mRNA displayed no significant changes in the different phases of brain development. Neurons of various brain regions including the neocortex and the hippocampus were identified as the main cell type expressing both TLR7 and TLR9 in the developing brain. Taken together, our data reveal specific expression patterns of distinct TLRs in the developing mouse brain and lay the foundation for further investigation of the pathophysiological significance of these receptors for developmental processes in the central nervous system of vertebrates.

## Introduction

Toll was identified as a regulator of the dorsoventral axis polarity during embryogenesis of *Drosophila melanogaster* [1]. Subsequently, it was found to play a key role in the fly's immune response [2]. The orthologs of Toll in vertebrates, the Toll-like receptors (TLRs), are innate immune receptors that detect both highly conserved pathogen-associated molecular patterns and host-derived molecules released during tissue damage. All of the known TLRs bind to adaptor proteins, among which myeloid differentiation primary response protein 88 (MyD88) and Toll/IL-1 receptor (TIR) domain-containing adaptor inducing IFN-β (TRIF) are key to subsequent activation of the intracellular signaling cascades that ultimately cause the release of inflammatory molecules from immune cells [3]. TLRs are abundantly expressed in the peripheral immune system but are also present in the central nervous system (CNS) where they mediate both immunological functions and responses to injury [4], [5]. Microglia, the major immune cells of the brain, express the full repertoire of TLRs, but some of these receptors are also present in neurons. However, the functional implications of TLR expression in neurons are not fully understood yet. Experimental evidence suggests that distinct TLRs regulate neural plasticity and development in neurons [6]. For example, TLR3 inhibits neural progenitor cell proliferation in the embryonic mouse telencephalon and regulates axonal growth [7], [8]. In addition, TLR8 is involved in injury and neurite outgrowth associated with neural development [9]. Finally TLR2 and TLR4 play a role in adult neurogenesis of the hippocampus [10], [11]. A role of TLRs in brain development and morphogenesis of vertebrates beyond these specific settings is mostly unknown. Although endogenous ligands of TLRs including extracellular matrix proteins and heat shock proteins exist, the host-derived molecules stimulating TLRs in the context of CNS development remain unidentified [12].

In this study we systematically characterize the expression of TLR1–9 as well as of MyD88 and TRIF during pre- and postnatal development of the mouse brain. CNS neurons of mouse embryos and newborns upregulate TLR7 transiently around birth. In contrast, expression of TLR9 increases constantly during late embryogenesis and postnatal stages until adult levels are reached. Although not found being statistically significant, TLR1, TLR3, and TLR6 expression also tend to follow a development-dependent pattern. Expression of other TLR family members and of MyD88 and TRIF was low and did not change significantly during the analyzed time period. The fact that distinct TLRs exert specific expression patterns over time in the developing mouse brain suggests a physiological relevance of specific TLRs in vertebrates in brain development.

**Figure 1:**
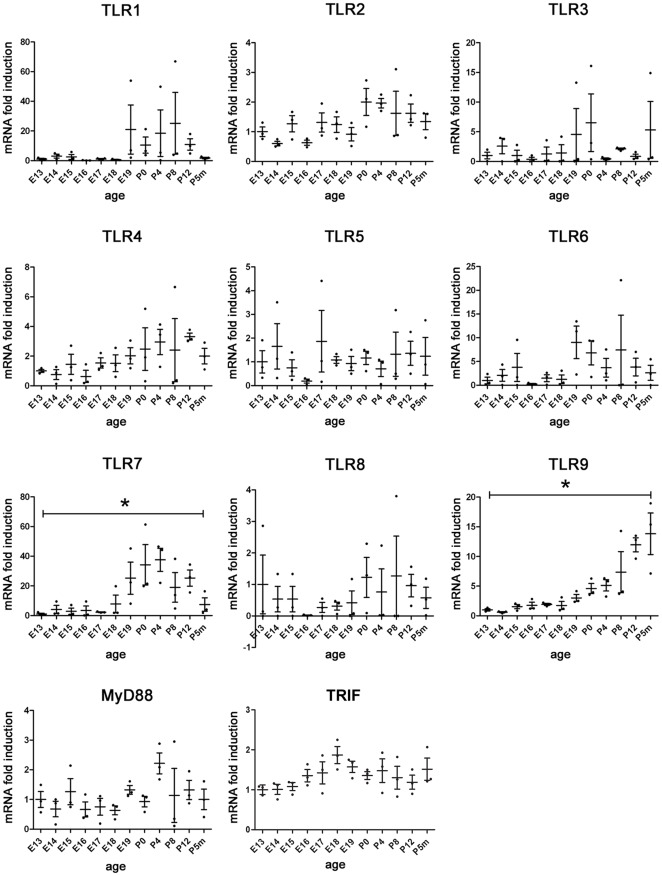
Expression of TLR1–9 mRNA, MyD88 mRNA, and TRIF mRNA in the developing mouse brain. Brain homogenates from C57Bl/6J mice at different embryonic (E), postnatal (days after birth, P), and adult (age of months, Pm) stages, as indicated, were assayed by quantitative real-time PCR using primers specific for TLR1–9, MyD88, and TRIF. Relative quantification was assessed by using the formula 2^−ΔCT^ and by normalizing the amount of the target gene to the housekeeping gene GAPDH, whose expression levels were not significantly altered during brain development (data not shown). Mean value of E13 was set to 1-fold induction, and mean values of all other ages were related to E13. Results are presented as mean +/− SEM. Statistical analysis was performed using the Kruskal-Wallis one-way analysis of variance with *p<0.05.

**Figure 2:**
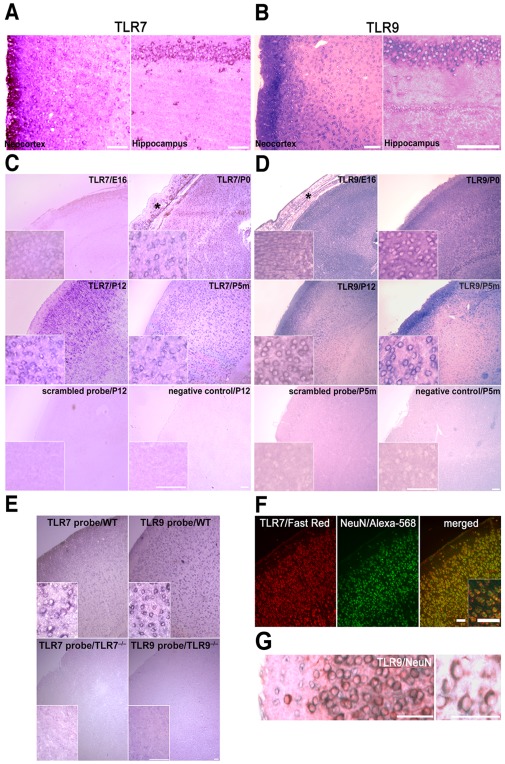
Time-dependent expression of TLR7 mRNA and TLR9 mRNA in neurons of the developing brain. *In situ* hybridization (NBT/BCIP) of the C57Bl/6J cerebral neocortex and hippocampus on P12 (**A**, **B**) and of the C57Bl/6J cerebral neocortex of different ages (**C**, **D**), as indicated, using probes specific for TLR7 (**A**, **C**), TLR9 (**B**, **D**), or a scrambled control probe. Insets in **C** and **D** display images of the neocortex with higher magnification. (**E**) *In situ* hybridization (NBT/BCIP) of the P5m-old C57Bl/6J (WT), TLR7KO, and TLR9KO neocortex using probes specific for TLR7 or TLR9, as indicated. *In situ* hybridization (Fast Red) of adjacent brain sections positive for TLR7 (on P12, **F**) or TLR9 (on P5m, **G**) co-immunostained with NeuN antibody and visualized by an Alexa-568-conjugated secondary antibody (**F**) or by DAB staining (**G**), respectively. Scale bar, 50 µm. (*) marks the skull.

## Results

### mRNA expression of TLRs, in particular TLR7 and TLR9, in the mouse brain correlates with different developmental stages

To systematically characterize the expression of the members of the TLR family in the mammalian developing brain, brains from mice at 12 different stages of development were analyzed by quantitative real-time PCR using primers specific for TLR1–9, MyD88, and TRIF. Time points of brain sampling were embryonic days E13–19, P0, postnatal days P4, P8, and P12 as well as the age of 5 months (P5m) (Fig. 1). Expression of TLR7 and TLR9 mRNA displayed significant changes during the analyzed periods. In detail, TLR7 mRNA expression was detectable in the brain as early as E13, increased on E18, and reached an almost 40-fold induction on P4 compared to embryonic stages. After P4 TLR7 expression decreased during the postnatal period to the low abundance found in the adult brain. Expression of TLR9 mRNA increased continuously during embryogenesis and postnatal stages until a 13-fold induction compared to embryonic stages was reached at an adult age. Although not statistically significant, expression of several other TLRs also seemed to follow a specific development-dependent pattern. For example, whereas expression of TLR1 tended to be increased around birth and on P12, TLR3 expression seemed to reach higher levels on P0 and P8 compared to embryonic stages. TLR6 expression revealed by trend an increased level around birth. In contrast to these receptors, expression of TLR2, TLR4, TLR5, TLR8, MyD88, and TRIF mRNA was low during the whole analyzed period.

**Figure 3:**
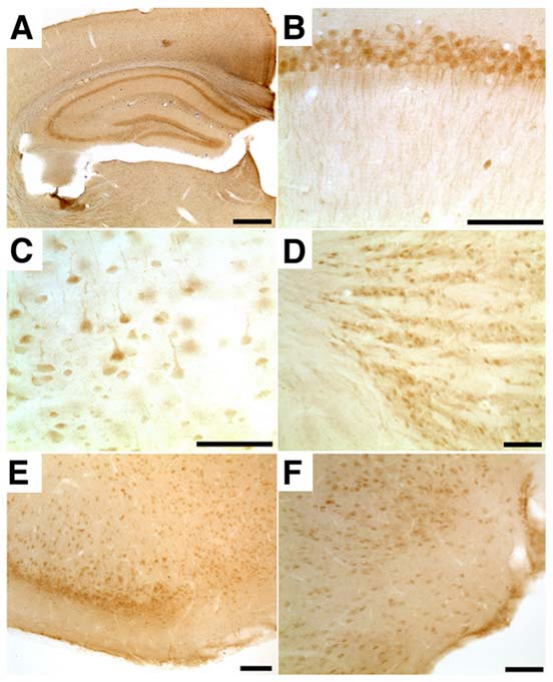
TLR7 protein is expressed in various regions of the mouse brain. Brain sections of P12 C57Bl/6J mice were incubated with an antibody against TLR7 and stained with DAB (**A**, overview). Cutouts from various brain regions including hippocampus (**B**), parietal neocortex (**C**), reticular thalamic nucleus (**D**), piriform cortex (**E**), and posterio-medial cortical amygdala (**F**) are shown. Scale bars, **a**: 500 µm; **b**, **c**, **d**, **f**: 50 µm, **e**: 100 µm.

**Figure 4:**
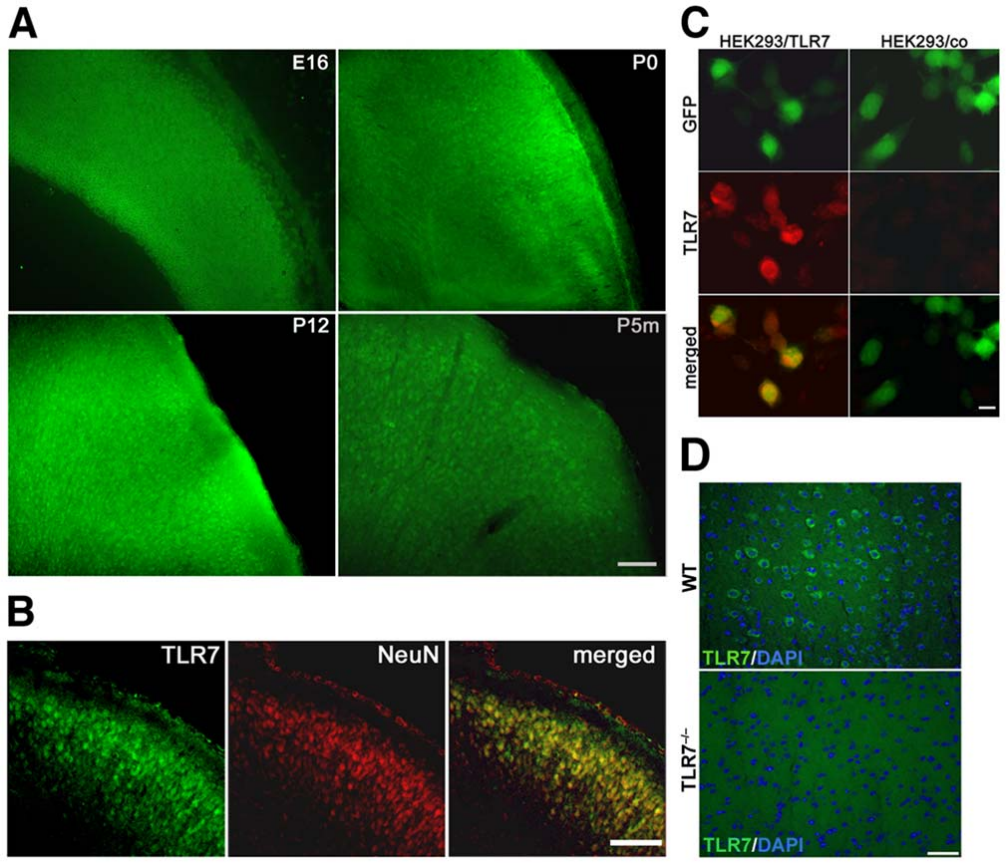
Expression of TLR7 protein in the developing mouse brain. (**A**) Immunostaining of the neocortex from C57Bl/6J mice with an antibody against TLR7 at different developmental stages, as indicated. Scale bar, 100 µm. (**B**) Adjacent brain sections from P12 mice were co-immunostained with antibodies against TLR7 and NeuN and analyzed by confocal sequential microscopy. Scale bar, 50 µm. (**C**) HEK293 cells transfected with pCAG-TLR7-IRES-eGFP and pCAG-IRES-eGFP were used as positive and negative control for immunostaining with the anti-TLR7 antibody, respectively. (**E**) Brain sections from P12 C57Bl/6J and TLR7KO mice were immunostained with the antibody against TLR7 named above and DAPI. Scale bar, 50 µm.

### TLR7 and TLR9 mRNA are expressed in neurons of the developing mouse brain

Having observed that expression of TLR7 and TLR9 mRNA is regulated during brain development, we investigated in which brain regions and in which cell types of the developing brain TLR7 and TLR9 are expressed. To this end, brain sections from mice of embryonic, perinatal, and adult stages were analyzed by *in situ* hybridization using specific probes for TLR7 and TLR9. Both TLR7 (Fig. 2A) and TLR9 (Fig. 2B) mRNA were readily detectable in various regions of the murine brain, including the hippocampus and the neocortex. In keeping with our findings using the quantitative real-time PCR analysis described above, TLR7 mRNA expression increased from embryonic to postnatal stages and then decreased to lower expression levels in the adult brain (Fig. 2C). Likewise, TLR9 mRNA expression continuously increased during embryonic and postnatal stages until adult levels were achieved (Fig. 2D), resembling the PCR results described above. Specificity of the employed TLR7 and TLR9 *in situ* hybridization probes were confirmed by including negative control probes with a scrambled sequence (Fig. 2C,D) and by analyzing comparable sections from TLR7- and TLR9-deficient mice, respectively (Fig. 2E). Parallel immunostaining of adjacent sections of the neocortex with a NeuN antibody identified most of the TLR7-positive (Fig. 2F) and TLR9-positive (Fig. 2G) cells as neurons.

**Figure 5:**
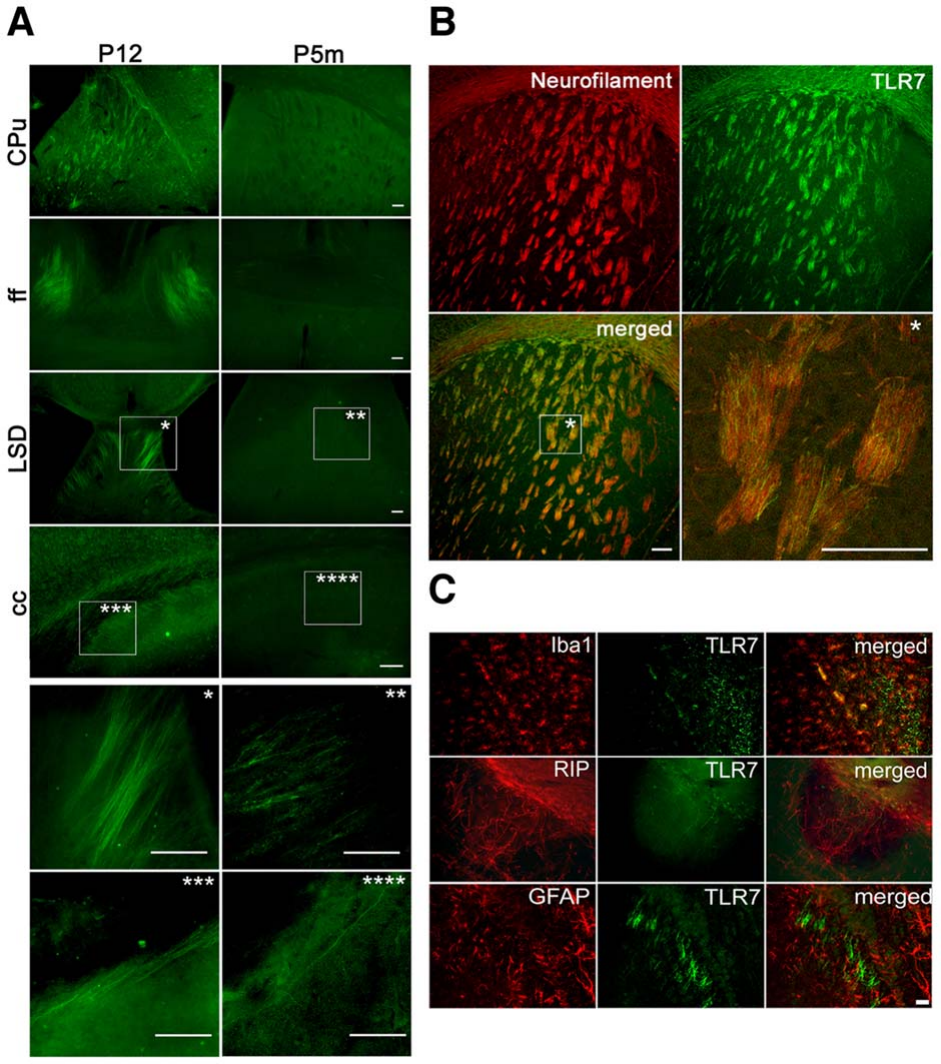
Axons express TLR7 during development of the brain. (**A**) Immunostaining of brain sections from C57Bl/6J mice on P12 and P5m using an antibody against TLR7. An overview and higher magnification images (*, **, ***, ****) displaying TLR7 expression in various brain regions are shown. CPu: caudate putamen; **LSD**: dorso-**lateral** septum; ff: fimbria, fornix; cc: corpus callosum. (**B**) Brain sections from P12 mice were co-immunostained with antibodies against TLR7 and neurofilament and subsequently were analyzed by confocal sequential microscopy. An overview and a higher magnification image (*) displaying TLR7 expression in the striatum are shown. Scale bar, 100 µm. (**C**) Brain sections from P12 mice were co-immunostained with antibodies against TLR7 and GFAP, RIP, or Iba1 to mark astrocytes, oligodendrocytes, and microglia in the corpus callosum, respectively. Subsequently, immunostained brain sections were analyzed by confocal sequential microscopy. Scale bar, 10 µm.

**Figure 6:**
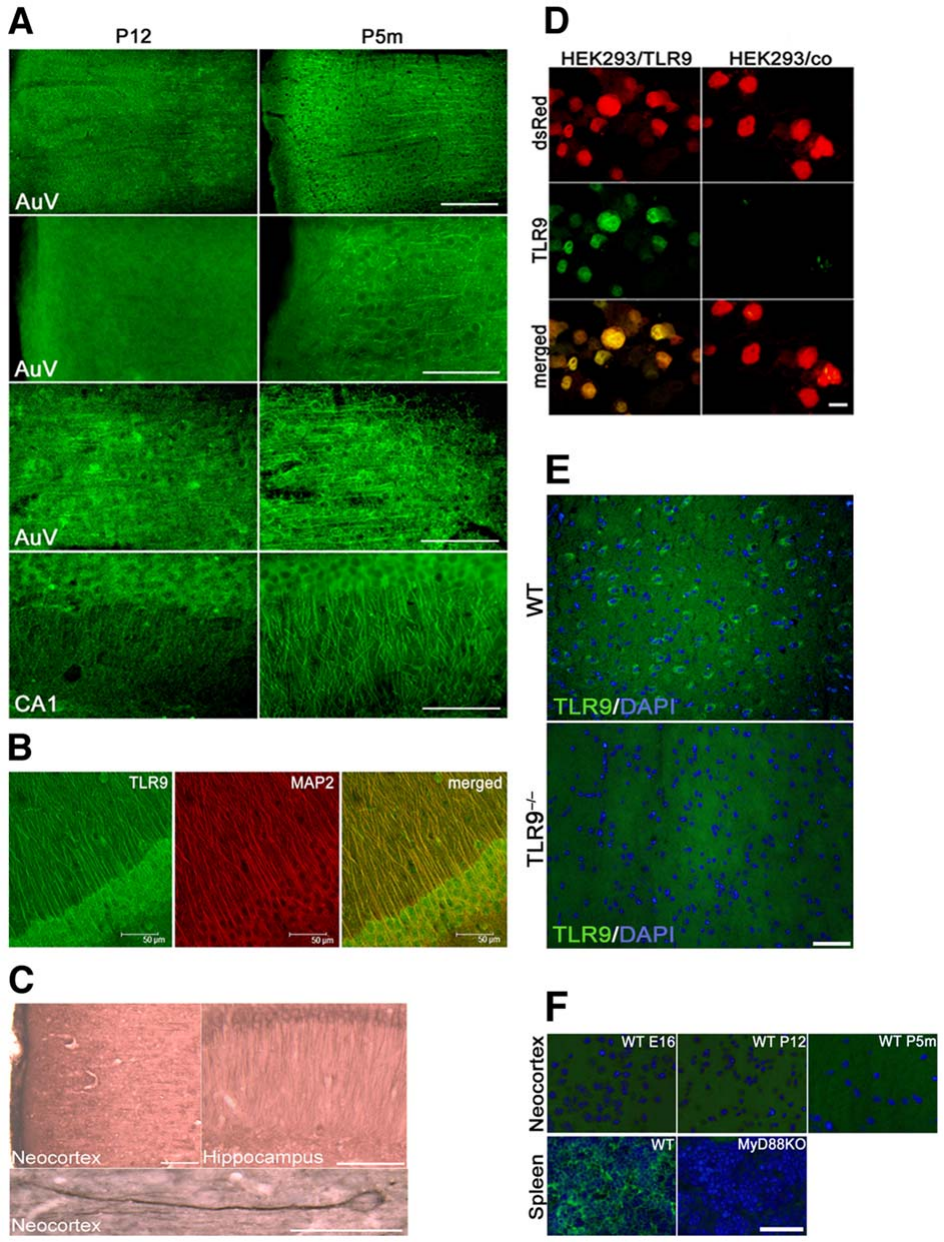
Expression of TLR9 protein in the developing brain. (**A**) Immunostaining of the neocortex and hippocampus with an antibody against TLR9 on P12 and P5m. AuV: secondary auditory cortex, CA1: cornu ammonis 1. Scale bar, 100 µm. (**B**) Brain sections from P5m C57Bl/6J mice were co-immunostained with antibodies against TLR9 and MAP-2, and the hippocampus was analyzed by confocal sequential microscopy. (**C**) Adjacent brain sections were incubated with an antibody against TLR9 and stained with DAB. Scale bar, 100 µm (upper images) and 50 µm (lower image). (**D**) HEK293 cells co-transfected with TLR9 and a DsRed vector served as positive control for immunostaining with the anti-TLR9 antibody. Transfection with the DsRed vector alone was used as negative control. (**E**) Brain sections from P5m-old C57Bl/6J (WT) and TLR9KO mice were immunostained with the antibody against TLR9 named above and DAPI. (**F**) Brain sections from E16-, P12-, and P5m-old WT mice were immunostained with an antibody against MyD88 and with DAPI. Spleen sections from P5m-old WT and MyD88KO mice served as positive and negative control for immunostaining with the anti-MyD88 antibody, respectively. Scale bar, 50 µm.

### Expression of TLR7 protein in CNS neurons is increased perinatally

Immunohistochemistry using a TLR7 antibody revealed abundant expression of TLR7 protein in various regions of the brain (Fig. 3). TLR7 protein was mainly detected in neurons, as judged by morphological analysis (Fig. 3). Expression of TLR7 protein in the neocortex increased during brain development and reached a peak during early postnatal phases (Fig. 4A). Confocal microscopy of brain sections co-immunostained with a NeuN antibody displayed expression of TLR7 protein in neurons of the neocortex, particularly in those associated with the layers II and III, as expected from the RNA studies described above (Fig. 4B).

Specificity of the anti-TLR7 antibody was confirmed by demonstrating its reactivity with HEK293 cells transfected with TLR7 (Fig. 4C) and by analyzing comparable brain sections from TLR7-deficient mice (Fig. 4D).

### Expression of TLR7 in axons in the developing brain

TLR7 protein was mainly detected in neuronal cell bodies of the neocortex and the hippocampus, but isolated elongated structures, which were judged as axons morphologically, in various brain regions also stained positive for TLR7 at different developmental stages. In particular, these TLR7-positive axons, partially bundled as tracts, were present in the caudal putamen, dorso-lateral septum, fimbria-fornix system as well as the corpus callosum (Fig. 5A). As observed for neuronal cell bodies, expression of TLR7 protein in these axons varied depending on the developmental stage. Whereas no or low levels of TLR7-positive axons were observed in the respective brain regions of sections derived from adult mice, marked expression was found in the same regions of sections from mice at early postnatal stages (Fig. 5A). Brain sections from P12 mice were co-immunostained with antibodies against TLR7 and neurofilament. Analysis by confocal microscopy revealed that the axons positive for TLR7 did not co-localize with the neurofilament antibody, but were instead nestling between neurofilament-positive axons, which did not express TLR7. However, some very few punctiform structures positive for both neurofilament and TLR7 antibody were observed and interpreted as crossovers of the two named groups of axons (Fig. 5B). Likewise, immunostaining of the brain sections with the MAP-2 antibody, an established marker for dendrites, did not result in co-localization with TLR7 (data not shown). In order to rule out that these isolated extended structures were extensions from other CNS cells than neurons, brain sections from P12 mice were co-immunostained with antibodies against TLR7 and GFAP, RIP, or Iba1 antibodies to mark astrocytes, oligodendrocytes and microglia, respectively (Fig. 5C). As expected, TLR7 in the axon-like structures did not co-localize with any of the glial markers named above. In contrast, cell bodies of microglia were positive for both TLR7 and Iba1 (Fig. 5C), as expected.

In summary, TLR7 is expressed in neurons and neurofilament-negative axons in the developing mouse brain, particularly in the early postnatal phase.

### Expression of TLR9 protein in neurons of the CNS increases with advancing age

Immunohistochemistry revealed an increased expression of TLR9 protein in the hippocampus and the neocortex of adult mouse stages compared to postnatal stages (Fig. 6A), resembling the PCR findings reported above.

Co-immunostaining of brain sections from adult animals with a MAP-2 antibody and subsequent analysis by confocal microscopy localized TLR9 protein to neuronal cell bodies and dendrites in the hippocampus (Fig. 6B). In addition, morphological analysis of the neocortex and hippocampus of adjacent brain sections by DAB staining confirmed expression of TLR9 protein in axons (Fig. 6C).

Specificity of the anti-TLR9 antibody was validated by demonstrating its reactivity with HEK293 cells transfected with TLR9 (Fig. 6D) and by analyzing comparable brain sections from TLR9-deficient mice (Fig. 6E).

Since both TLR7 and TLR9 signal through MyD88 [3], protein expression of this adaptor protein in the CNS was investigated by additional immunostainings of brain sections derived from different developmental stages (Fig. 6F). No distinct signal was detected in brains from embryonic, postnatal, or adult mice after treatment with an antibody directed against MyD88. In contrast, spleen cells, which served as positive control, displayed strong expression of MyD88, as expected. Specificity of the employed antibody was confirmed by analyzing comparable spleen sections from MyD88-deficient mice, in which no signal for MyD88 was detected after immunostaining with the named antibody (Fig. 6F).

## Discussion

TLRs mediate both innate and adaptive immunity. They were extensively studied as pathogen-recognition receptors in vertebrates so far. Toll in *Drosophila melanogaster* is involved in developmental processes that control the dorsoventral axis of the fruit fly embryo, synaptogenesis, and axon path finding [1], [13]. Such non-immune functions for TLRs are mostly unknown in mammals, although these receptors are evolutionary highly conserved across species, and evidence emerges that traditional immune molecules may unfold distinct functions in neuronal processes [14].

We show that distinct TLR family members are expressed in specific temporal patterns during development of the mouse brain. Expression profiles of TLR7 and TLR9 revealed time-dependent regulation, whereas expression levels of the other tested TLRs and the adaptor molecules MyD88 and TRIF did not change significantly in the developing brain. Neurons of various brain regions, in particular those in the neocortex and hippocampus, were the main CNS cell type expressing TLR7 and TLR9 during the analyzed period. Although the functional relevance of these receptors for the developing CNS remains unknown, previous work suggests a role for other TLR family members in various aspects of neural development [15]. For example, TLR2 and TLR4 in neural progenitor cells (NPC) may have an impact on their proliferation and differentiation, and mice lacking TLR2 display impaired neurogenesis in the hippocampus [10]. TLR3 was described as a negative regulator of NPC proliferation and to control axonal growth [7], [8], and TLR8 is involved in apoptosis of axons and neurons in the developing brain [9]. Thus, TLRs may exert various functions in different developmental contexts. Despite the reports cited above, only TLR7 and TLR9 were significantly regulated during CNS development in our work. This discrepancy might be explained, at least in part, by the use of whole forebrains in our study, whereas distinct CNS regions were investigated selectively in the previous works. We may have missed subtle changes of expression patterns in our more holistic analysis. In addition, differences regarding the analyzed time points and mouse strains have to be considered when comparing studies on TLR expression in developmental processes. For example, whereas C57Bl/6J mice were used in our study, Ma and colleagues have employed Swiss Webster mice in their work. Also, the exact developmental stages analyzed in our study differ from the time points chosen by the named group [9].

Our data demonstrate that TLR7 is not only expressed in neuronal cell bodies but also in axons that were present in various brain regions, partly bundled to tracts and partly nestled between axons, which lacked TLR7. Although these structures were morphologically judged as axons, they did not stain with established antibodies directed against neurofilament or myelin [16], [17]. We hypothesize that these structures represent axonal subtypes that appear only at specific developmental stages and escape detection with established axonal markers. In accordance with this hypothesis, these TLR7-positive axons were mainly observed in the early postnatal phase important for CNS morphogenesis but were absent or much less frequent in the adult brain. Toll is involved in axon path finding in the fruit fly embryo [13], and several reports discuss a role for TLRs in neurite outgrowth in vertebrates [8], [9]. It is therefore possible that the TLR7-positive axons observed in our studies are involved in axon path finding during development of the mouse brain.

TLR7 and TLR9 in immune cells recognize single-stranded RNA and unmethylated CpG DNA from pathogens [3], respectively, but the putative ligand for these TLRs in the context of brain development remains unknown. Importantly, TLRs in immune cells can respond to alterations in their immediate vicinity by recognizing host-derived molecules including matrix components, their degradation products, or molecules released from injured cells [18], [19]. It is conceivable that TLR-dependent control of developmental processes in the CNS such as neurogenesis, neurite outgrowth, or axon path finding occur as part of a physiological response to tissue injury. Later in life, pathways involved in development may be reactivated in response to injury. One possibility is that TLR7 and TLR9 in the developing brain are stimulated by nucleic acids derived from the host's tissue instead by pathogen-associated RNA or DNA in infectious situations. The exact source and localization of such endogenous ligands during brain development remains to be determined. It is unknown whether the respective ligand is released from the neurite itself and acts through an autocrine mechanism or originates from the target structure, thereby guiding neurons expressing the matching TLR. Since TLR7 and TLR9 localize to endosomes, at least in immune cells [3], the involved ligands would have to enter the neuron to bind to the respective TLR. Also the intracellular signaling cascade involved in TLR signaling during development in vertebrates remains to be elucidated. In contrast to TLR7 and TLR9, expression of the intracellular adaptors MyD88 and TRIF was unaltered during brain development in our study. This result is consistent with the previous finding that the TLR8-mediated response in neurons does not involve the canonical TLR-NF-κB signaling pathway in the context of brain development [9]. In accordance with this, mice deficient of both MyD88 and TRIF and therefore lacking the established signaling pathways downstream of TLRs do not seem to be grossly impaired in terms of CNS development [20]. One candidate signaling molecule among others is the adaptor molecule sterile-alpha- and armadillo-motif-containing protein (SARM), which is preferentially expressed in neurons and regulates neuronal survival [21].

The finding that TLR7 and TLR9 are prominently and time-dependently expressed in the developing brain must also be discussed in the context of infections. Perinatal expression of TLR7 in neurons and glia may influence the receptiveness and the responsiveness of the developing brain to viral infections, which in turn may play a role in the etiology of neurodevelopmental disorders including autism and schizophrenia [22], [23], [24]. The impact of TLR activation on developmental processes may depend on the state of the local environment, i.e. physiological versus pathological conditions. This hypothesis is supported by the fact that TLRs are expressed in glial cells in the neurogenic niches of the adult brain, and immune responses are suggested to affect hippocampal neurogenesis [25], [26], [27].

In summary, we show that TLR7 and TLR9 undergo significant regulation during development of the murine brain, whereas no corresponding changes were observed for the expression of other TLR family members. This finding supports a role for TLRs in brain development in vertebrates in general and for a specific role for TLR7 and TLR9. However, mice deficient for a single TLR or combinations of two TLRs are viable. Although their brains have not been investigated in detail on a microanatomic level, no major impairment in CNS development resulting in morphogenetic failures in these animals was described [28], [29]. Thus, a single TLR may not be irreplaceable during development of the CNS. Since the TLR family exerts high redundancy in the immune system [30], allowing various TLRs to assume the role of other TLR family members, further investigation of animals lacking whole functional groups of TLRs, such as TLR3, TLR7, and TLR9 might be required to understand the TLRs' role in the specific context of development. Also, other pathways downstream of TLRs besides the established ones that signal through MyD88 or TRIF may have to be considered and identified to gain deeper insight into the pathophysiological significance of TLRs in the development of the vertebrate brain.

## Materials and Methods

### Mice and cell lines

C57Bl/6J (WT) mice were purchased from Charles River, Sulzbach, Germany. TLR7 knock out (KO), TLR9KO, and MyD88KO mice were generously provided by Dr. S. Akira (Osaka University, Department of Host Defense, Osaka, Japan). All animals received humane care according to institutional criteria. All animal procedures were approved by the Landesamt für Gesundheit und Soziales Berlin (LAGeSo registration T0046/02). HEK293 cells were provided by American Type Culture Collection, Manassas, VA, and were cultured in DMEM supplemented with 10% heat-inactivated FCS and penicillin-streptomycin. Cells were grown at 37°C in humidified air with 5% CO_2_.

### Immunocytochemistry and immunohistochemistry

Immunostainings were performed as described previously [19]. The following primary antibodies were used: anti-neuronal-specific nuclear protein NeuN, anti-neurofilament, anti-microtubule-associated protein (MAP-2), anti-glial fibrillary acidic protein (GFAP), anti-MyD88 (C-terminus), and anti-RIP (anti-oligodendrocytes, clone NS-1); all purchased from Millipore, Billerica, MA. anti-TLR7 and anti-TLR9 were purchased from Imgenex, San Diego, CA; anti-Iba1 was from Wako, Neuss, Germany. Fluorescence microscopy was performed using an Olympus BX51 microscope and a confocal laser scan Leica TCS SL microscope with sequential analysis. For DAB (3,3′-diaminobenzidine) staining incubation with the primary antibody was followed by three washes in 0.1 M PB and incubation at room temperature with the appropriate biotinylated secondary antibody (Vector Laboratories, Burlingame, CA) diluted 1∶250 in 0.1 M PB for 1 h. Visualization of antibody binding by DAB staining was performed using the ABC Standard Kit (Vector Laboratories) with DAB/H_2_O_2_ as substrates following the manufacturer's suggestions.

### In situ hybridization

*In situ* hybridization of mouse brain cryostat sections of different ages, as indicated, using probes specific for TLR7 (5′-TAATCACATCCACTTTTTCATC-3′) or TLR9 (5′-GGTCCAGTTAAAGAAAGATAGGT-3′) and a scrambled control probe (5′-GTGACACGTCTATACGCCCA-3′) synthesized by Exiqon, Vedbaek, Denmark, was visualized by Fast Red or NBT/BCIP and was performed as described previously [31].

### Quantitative real-time RT-PCR

Native brains were isolated from C57BL/6J mice and tissue of the telencephalon and diencephalon of the same developmental stage (E13, E14, E15, E16, E17, E18, E19, P0, P4, P8, P12, P5m, *n* = 3–5 per age group) were pooled and snap-frozen in liquid nitrogen. mRNA was prepared using the Absolutely RNA Miniprep Kit (Stratagene, La Jolla, CA) and reversely transcribed to cDNA by a murine leukemia virus reverse transcriptase (Gibco, Karlsruhe, Germany). Expression of Toll-like Receptors 1–9, MyD88, TRIF, and GAPDH was analyzed by quantitative real-time PCR using specific primers for the respective candidate molecule and the SYBR Green dye as the fluorescent reporter (*n* = 3, Super Array, Frederick, MD). PCR reactions were performed with 1 µg of cDNA using an ABI PRISM 7500 Fast Lightcycler (Applied Biosystems, Foster City, CA). Relative quantification was assessed by using the formula 2^−ΔCT^ and by normalizing the amount of the target gene to the housekeeping gene GAPDH.

RNA degradation was ruled out by testing the respective RNA preparations on denaturing agarose gels. All gel runs resulted in sharp 18S and 28S rRNA bands. Also, the 28S rRNA band was constantly approximately twice as intense as the 18S rRNA band indicating that the tested RNA was intact (data not shown).

### Statistical analysis

Quantitative real-time RT-PCR data were analyzed using the Kruskal-Wallis Test (GraphPad Prism 5.00, GraphPad Software, San Diego, CA). Differences were considered statistically significant with *p*<0.05.
